# Pelvic Gastrointestinal Stromal Tumor (GIST) Mimicking Ovarian Cancer in a Kidney–Liver Transplant Recipient Under Chronic Immunosuppression

**DOI:** 10.1155/crom/2158098

**Published:** 2026-02-04

**Authors:** Oyepeju F. Abioye, Rachel DiLeo, Rahim Jiwani, Whitney Rich, Sharon Liang, Dulabh Monga

**Affiliations:** ^1^ Department of Internal Medicine, Allegheny Health Network, Pittsburgh, Pennsylvania, USA, ahn.org; ^2^ Division of Medical Oncology, Allegheny Health Network Cancer Institute, Pittsburgh, Pennsylvania, USA; ^3^ Department of Pathology, Allegheny Health Network, Pittsburgh, Pennsylvania, USA, ahn.org

## Abstract

Gastrointestinal stromal tumors (GISTs) are rare mesenchymal neoplasms that predominantly originate in the gastrointestinal tract. Extra‐gastrointestinal GISTs can occur in atypical locations such as the pelvis, which may mimic gynecologic malignancies, creating diagnostic challenges. This case report presents a 39‐year‐old female with a history of Type 1 diabetes mellitus and prior kidney and liver transplantation who presented with progressive abdominal bloating and discomfort. Initial pelvic ultrasound revealed a large right adnexal mass (18.8 × 12.8 × 9.8 cm), suggestive of an ovarian mass. CT imaging confirmed a complex pelvic tumor exerting mass effect on surrounding organs, initially concerning for gynecologic malignancy. Following an unrevealing endoscopic evaluation, the patient underwent exploratory laparotomy with total abdominal hysterectomy, bilateral salpingo‐oophorectomy, and tumor debulking. Intraoperative findings included a large right pelvic mass with extensive adhesions, friable tumor implants, and mesenteric lymphadenopathy. Postoperative pathology confirmed a high‐grade GIST with epithelioid features, positive for DOG1 and CD117, with a Ki‐67 index exceeding 30%. Molecular testing identified a KIT Exon 9 mutation, leading to initiation of imatinib therapy. Overall, this case represents an extra‐GI/pelvic GIST that mimicked a primary ovarian neoplasm. We achieved a complete macroscopic cytoreduction (no gross residual disease) but explicitly note pT4 (due to intraoperative rupture), informing adjuvant KIT inhibition. This case emphasizes the diagnostic challenge of extra‐GI/pelvic GISTs mimicking ovarian tumors. Studies show that GISTs mimicking primary ovarian tumors (GIST‐OTs) typically occur in younger women, have lower recurrence rates (6.8% vs. 54.5% in metastatic ovarian GISTs), and achieve complete resection more frequently (> 90% vs. 57% in metastatic cases). Immunohistochemical profiling (DOG1 and CD117) and molecular testing are crucial for accurate diagnosis and treatment planning. Although imatinib remains the cornerstone of GIST management, dose adjustments based on specific mutations may be necessary, as patients with KIT Exon 9 mutations might benefit from higher dosing. Multidisciplinary approaches combining imaging, histology, and molecular profiling are essential for optimizing outcomes in these complex cases. This extra‐GI/pelvic GIST occurred under chronic posttransplant immunosuppression after renal and liver transplantation; as such, we highlight the transplant–oncology interface, notably, an elevated posttransplant cancer risk, rare but documented GIST after kidney transplant, and TKI–calcineurin‐inhibitor interactions that require coordinated management.

## 1. Introduction

Gastrointestinal stromal tumors (GISTs) are uncommon mesenchymal tumors originating predominantly in the gastrointestinal tract, particularly in the stomach and small intestine. Extra‐gastrointestinal GISTs, although rare, may occur in atypical locations such as the pelvis, where they can closely resemble gynecologic tumors. This case report highlights a patient whose large pelvic GIST presented similarly to a primary ovarian tumor (OT), complicating the diagnostic and therapeutic approach.

Solid‐organ transplant recipients have an approximately twofold to fourfold higher overall cancer risk than the general population, largely attributed to chronic immunosuppression and impaired immune surveillance [1, 2]. Although uncommon, GIST has been reported in transplant patients in the literature (≈16 cases collated in a recent review) [3]. Our case adds to this small body of evidence by describing an extra‐GI pelvic GIST in a patient with dual solid‐organ transplants. We also discuss transplant‐specific diagnostic and pharmacologic considerations relevant to this presentation.

## 2. Case Presentation

**Figure 1:**
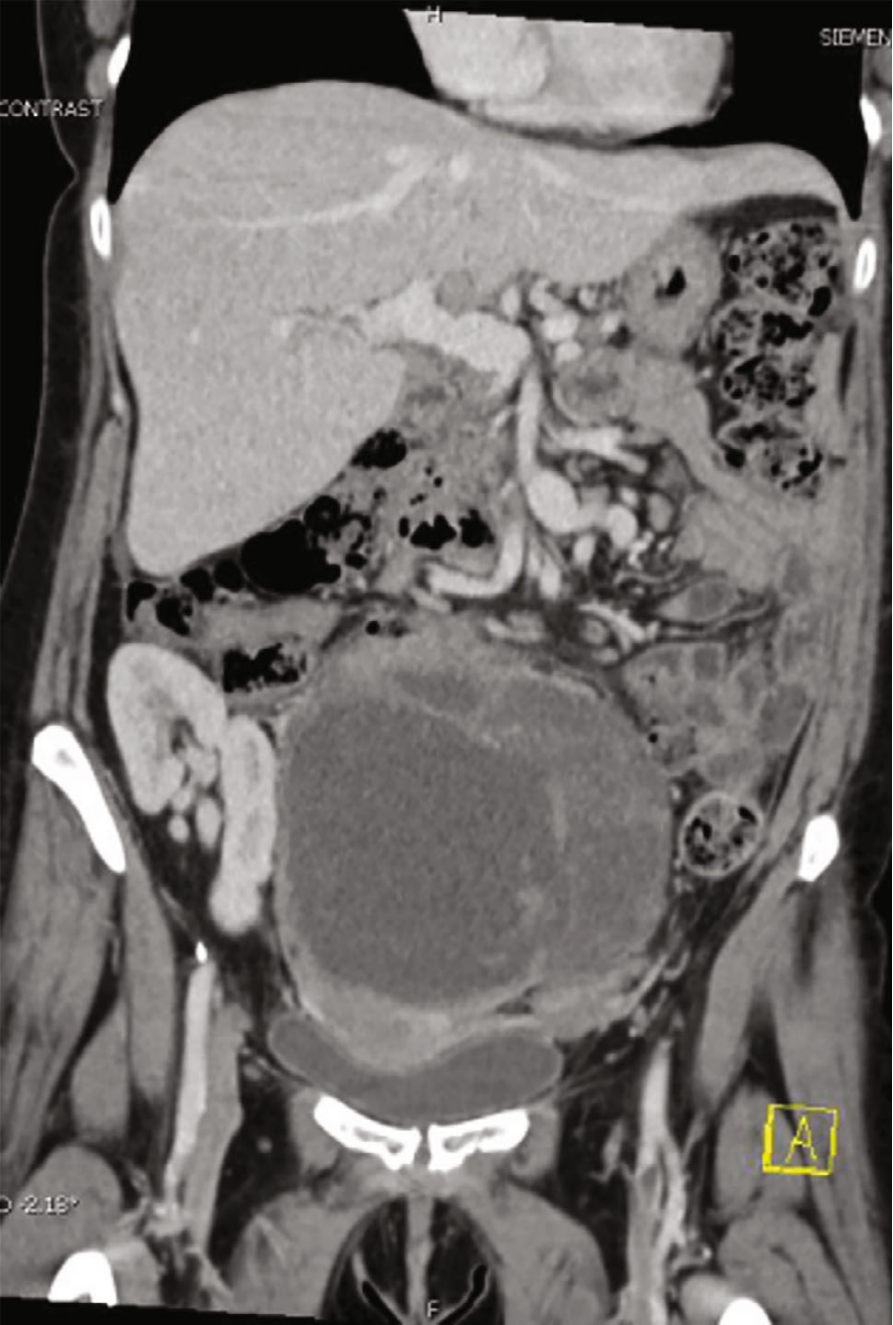
Coronal image of CT scan demonstrating large midline lower abdomen/pelvic mass with cystic areas measuring up to 19 × 12 × 13 cm, with mass effect on pelvic organs and bowel loops.

A 39‐year‐old female with a history of Type 1 diabetes mellitus (DM) complicated by end‐stage diabetic nephropathy, requiring kidney transplantation and immunosuppressive therapy, as well as a liver transplant due to alcoholic liver disease, presented with several months of progressive abdominal bloating and discomfort. Initial gynecological evaluation included a pelvic ultrasound, which revealed a large right adnexal mass measuring 18.8 × 12.8 × 9.8 cm, indicative of a possible ovarian involvement. Subsequent CT imaging demonstrated a complex pelvic tumor exerting mass effect on surrounding organs, concerning for a gynecologic malignancy (Figure 1).

The patient underwent in‐depth endoscopic evaluation to identify the primary tumor site, which was unrevealing. After counseling, the patient elected for a total abdominal hysterectomy–bilateral salpingo‐oophorectomy (TAH‐BSO); this was based on her strong family history, no desire for future fertility, and avoidance of reoperation. She subsequently underwent exploratory laparotomy, total abdominal hysterectomy, bilateral salpingo‐oophorectomy, and extensive tumor debulking. Intraoperative findings revealed a large right pelvic mass with extensive adhesions, as well as friable tumor implants on the rectal surface and small bowel, along with mesenteric lymphadenopathy. Postoperative pathology confirmed a diagnosis of high‐grade GIST with epithelioid features, positive for DOG1 and CD117, and a Ki 67 index greater than 30% (Figure 2). The tumor was classified as pT4 pN0, indicating no lymph node involvement. Margins were reported as no gross residual disease (macroscopic cytoreduction) without implying R0. Postsurgical complications included recurrent gastrointestinal bleeding, which was managed endoscopically. Molecular testing of the tumor identified a KIT Exon 9 mutation, leading to the initiation of imatinib therapy, which was subsequently titrated based on the patient′s tolerance.

Figure 2Cross‐section of pathology images showing: (a) interface of spindle neoplasm with areas of tumor cell necrosis and hemorrhage; (b) increased mitotic activity, including atypical mitotic figures; (c) DOG1 positivity; (d) CD 117 positivity.

**Figure:**
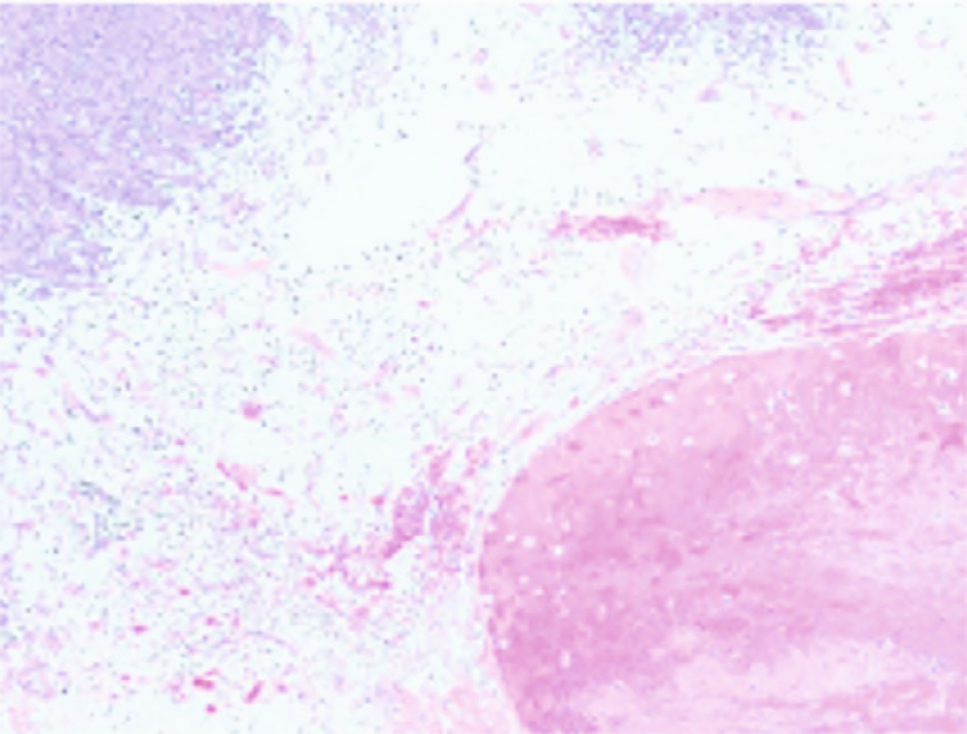
(a)

**Figure:**
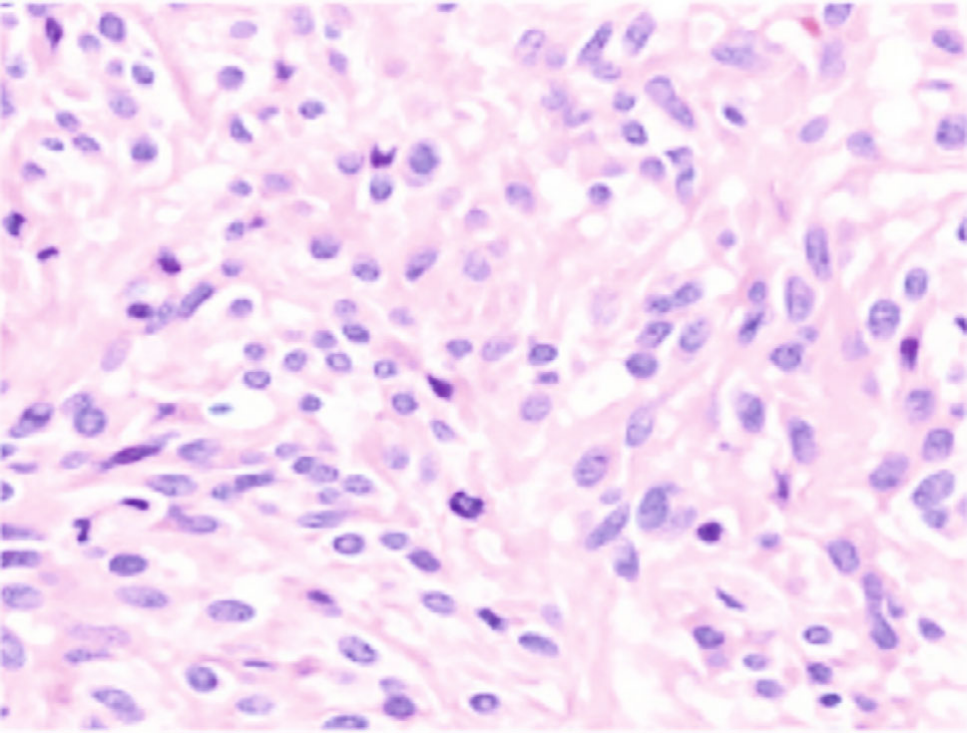
(b)

**Figure:**
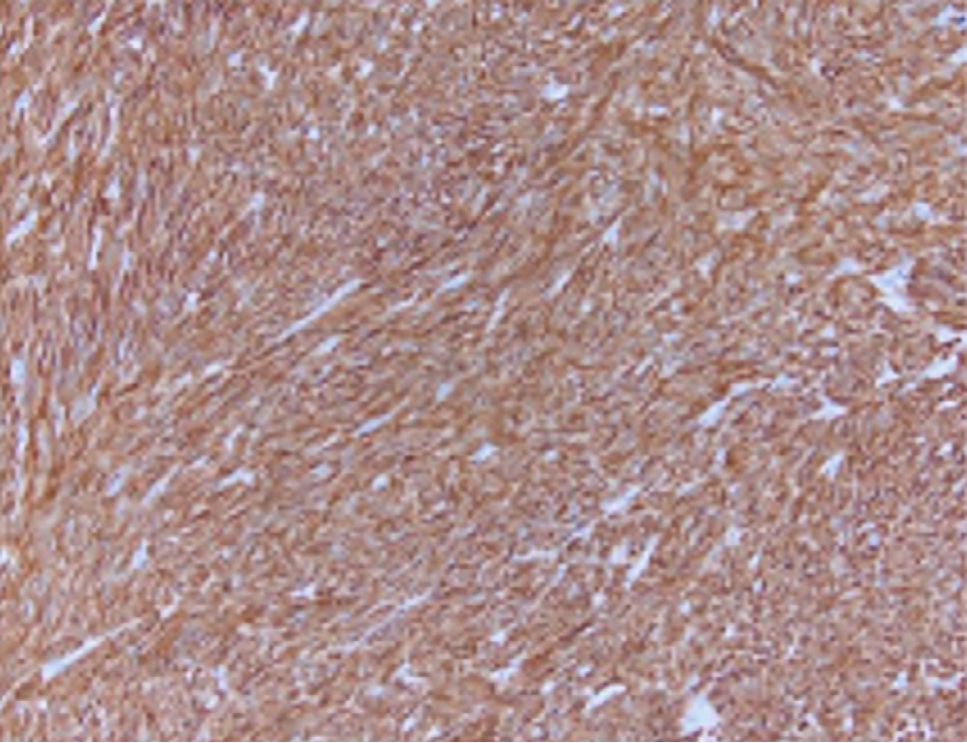
(c)

**Figure:**
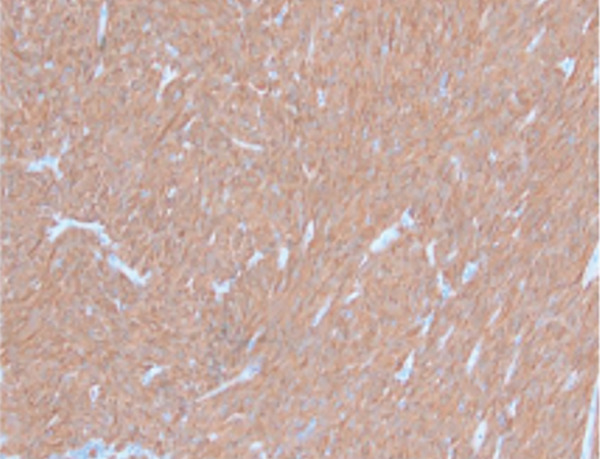
(d)

## 3. Discussion

GISTs are rare neoplasms, representing about 1% of gastrointestinal tumors yet making up 80% of mesenchymal cancers. Postulated to arise from the interstitial cells of Cajal, they can develop in standard digestive tract locations such as the stomach, small and large intestines, or less common sites like the omentum, mesentery, retroperitoneum, and pancreas. When arising from extra‐gastrointestinal sites like the ovaries, GISTs can mimic primary OTs or metastatic ovarian tumors (M‐OTs). This is particularly true when they present with significant mass effect or abdominal discomfort, and often lead to a misdiagnosis as gynecologic neoplasms [4–6]. Table 1 below provides a differential diagnosis of pelvic masses with an emphasis on GIST.

**Table 1:** Differential diagnosis of pelvic mass with emphasis on gastrointestinal stromal tumor (GIST).

**Category**	**Differential diagnoses**	**Key features**
Gastrointestinal tumors	Gastrointestinal stromal tumor (GIST, pelvic location)	Rare in the pelvis, usually arises from rectum or small bowel with exophytic growth. CD117 (c‐KIT) and DOG1 positive. Well defined, hypervascular mass on CT, often with necrosis or hemorrhage. May cause GI bleeding, anemia, or compression symptoms.
	Colorectal cancer	Irregular, firm mass, often with rectal bleeding, changes in bowel habits. Typically CK20 and CDX2 positive.
	Appendiceal neoplasm (e.g., mucinous neoplasm and carcinoid tumor)	May present as a cystic or solid mass, associated with pseudomyxoma peritonei if ruptured. Mucinous subtype can cause progressive peritoneal involvement.
	Diverticular abscess	History of diverticulitis, fever, localized pain, may show air‐fluid level on imaging. Resolves with antibiotics/drainage.

Gynecologic tumors	Ovarian cancer (e.g., sex cord stromal, germ cell, and epithelial carcinoma)	May present as a solid‐cystic mass with ascites. Elevated CA‐125. Frequently bilateral when advanced.
	Uterine leiomyoma, leiomyosarcoma	Firm, well‐circumscribed, associated with abnormal uterine bleeding. Leiomyosarcoma is more aggressive and lacks c‐KIT expression.
	Endometriosis with deep infiltrating nodules	Chronic pelvic pain, dysmenorrhea, often in reproductive‐age women. May appear as hemorrhagic cystic lesions on imaging.

Soft tissue/other mesenchymal tumors	Retroperitoneal sarcoma (leiomyosarcoma, liposarcoma, and desmoid tumor, etc.)	Large, slow‐growing, may compress adjacent structures. Leiomyosarcoma: smooth muscle origin, stains positive for SMA but negative for c‐KIT.
	Peritoneal carcinomatosis	Multiple nodular implants, often secondary to GI, ovarian, or peritoneal malignancies. May present with ascites.

Lymphomas	Lymphoma (pelvic nodal involvement)	Diffuse lymphadenopathy, B symptoms (fever, weight loss, night sweats). Typically PET‐avid.
Metastatic disease	Metastatic disease (gastric, pancreatic, colorectal, ovarian, or other primary tumors)	Often multiple lesions, history of primary malignancy. Imaging and biopsy needed for differentiation

Prognostic markers for ovarian GIST include younger age and nonmetastatic disease. GISTs mimicking OTs have been noted to occur more frequently in younger women and are less likely to be associated with elevated tumor markers, indicating a better prognosis compared with older women with M‐OTs, who may exhibit elevated markers and a worse prognosis [1]. Additionally, a systematic review found that patients with metastatic ovarian GISTs (GIST M‐OTs) had significantly higher recurrence and mortality rates compared with those with GISTs mimicking primary ovarian tumors (GIST‐OTs), with recurrence occurring in 54.5% of GIST M‐OT patients versus only 6.8% of GIST‐OT patients [4]. Prognosis was also noted to be significantly better in cases with complete resection, achieved in over 90% of GIST‐OTs but in only about 57% of GIST M‐OTs [4].

Immunohistochemical profile, particularly DOG1 and CD117 positivity, is crucial in differentiating GISTs from other tumors [7, 8]. Beyond immunohistochemistry, molecular profiling plays a key role [9]. Most GISTs harbor activating mutations in *KIT* (Exon 11 being the most common) or *PDGFRA*, which drive tumorigenesis and influence response to tyrosine kinase inhibitors, such as imatinib. Additionally, recurrence risk is significantly impacted by factors such as tumor size, location, and mitotic rate, with tumors larger than 5 cm or those exceeding 5 mitoses per 50 high‐power fields (HPF) carrying a higher risk of recurrence and metastasis [6]. For our patient′s resection status, to avoid ambiguity, we state complete macroscopic cytoreduction (no gross residual disease) rather than implying R0 margins, and we explicitly note pT4 due to intraoperative rupture, which drives high‐recurrence risk and informs the adjuvant KIT‐inhibitor strategy.

Beyond tumor‐intrinsic risk, the patient′s posttransplant immunosuppression added distinct diagnostic and therapeutic considerations. Solid‐organ transplant recipients have a ~2× overall increase in cancer risk versus the general population, attributed to chronic immunosuppression and impaired immune surveillance [1, 2]. GIST after kidney transplantation is rare but documented in the literature [3]. Although the mechanistic link between immunosuppression and mesenchymal tumorigenesis remains incompletely defined, the transplant setting may lower barriers to tumor emergence [3] and complicate recognition when masses arise in atypical locations (e.g., pelvis mimicking ovarian neoplasm). Maintaining GIST in the differential for large pelvic masses in transplant recipients is prudent, particularly when gynecologic markers are noncontributory. These data support interpreting our case within a recognized, if uncommon, transplant‐oncology context rather than implying causality.

Adjuvant treatment with the tyrosine kinase inhibitor (TKI) Imatinib remains the cornerstone of postoperative GIST management [10]. Additionally, imatinib remains the first‐line standard of care for metastatic GIST. However, dose adjustments may be necessary based on tolerability, as seen in our patient. Studies show that molecular profiling for mutations in KIT or PDGFRA genes is essential [9]. Although imatinib is typically administered at a standard dose of 400 mg daily, patients with different KIT mutations show variable responses. Patients with KIT Exon 9 mutations may benefit from higher dosing (800 mg daily), unlike those with KIT Exon 11 mutations, who generally respond well to standard dosing [11, 12]. This difference stems from the distinct molecular mechanisms by which these mutations affect imatinib binding and efficacy [11–13].

In the metastatic setting, a meta‐analysis of the EORTC‐ISG‐AGITG 62005 and SWOG S0033 Phase III trials demonstrated significant progression‐free survival (PFS) benefit for patients with KIT Exon 9‐mutated GIST when treated with high‐dose imatinib (800 mg daily) compared with standard dose (400 mg daily) [11]. However, this analysis did not demonstrate a statistically significant overall survival (OS) benefit. Another retrospective analysis [14] suggests that higher imatinib doses did not demonstrate improved survival outcomes compared with the lower dose in patients with KIT Exon 9 mutations. Conversely, in the adjuvant setting, the benefit of higher dosing for Exon 9‐mutated GIST remains less established, with current evidence not showing robust OS improvements [15]. As such, mutation‐specific and context‐dependent treatment approaches for GIST patients remain important. Additionally, two transplant‐specific issues warrant emphasis. First, drug–drug interactions: imatinib inhibits CYP3A4 and can increase tacrolimus exposure [16, 17], mandating trough monitoring and close collaboration with transplant pharmacology. Second, choice/adjustment of maintenance immunosuppression: meta‐analytic data suggest sirolimus regimens are associated with lower malignancy incidence overall [18], though potential trade‐offs (including mortality signals) require individualized assessment with the transplant team.

## 4. Conclusion

When GISTs in atypical locations, such as the pelvis, mimic OTs due to their presentation and mass effect on surrounding pelvic structures, it can lead to diagnostic delays, particularly if the initial presentation resembles gynecologic pathology. As such, atypically presenting GISTs require a multidisciplinary approach in their diagnosis and management to optimize patient outcomes. The ability to distinguish GISTs from ovarian malignancies and other pelvic tumors through a combination of imaging, histology, and molecular profiling is critical, as is careful management with TKIs in patients with extensive comorbidities. Understanding the unique presentation, diagnostic process, and multidisciplinary management is crucial for optimizing outcomes, particularly in complex cases involving significant coexisting conditions. In solid‐organ transplant recipients, chronic immunosuppression should be treated as a clinical context that can alter presentation, risk stratification, and pharmacology, potentially complicating management of pelvic GIST. For high‐risk/ruptured disease, guideline‐concordant adjuvant imatinib remains the reference approach when tolerated; when intolerance or KIT Exon 9 biology necessitates, a carefully monitored alternative is reasonable. Future reports that include agent class, trough levels, and graft function may clarify the immunosuppression–GIST relationship.

## Consent

The patient′s consent was obtained.

## Conflicts of Interest

The authors declare no conflicts of interest.

## Funding

No funding was received for this manuscript.

## Data Availability

The data that support the findings of this study are available from the corresponding author upon reasonable request.
